# The Coexistence of the Superficial Brachial Artery With the Common Origin of the Posterior Circumflex Humeral Artery and the Deep Brachial Artery

**DOI:** 10.7759/cureus.45903

**Published:** 2023-09-25

**Authors:** Christos Koutserimpas, George Tsakotos, Maria Piagkou, George Triantafyllou, Trifon Totlis, Chrysovalantis Mariorakis, Vasileios Karampelias, Konstantinos Natsis

**Affiliations:** 1 Orthopaedics and Traumatology, "251" Hellenic Air Force General Hospital of Athens, Greece, Athens, GRC; 2 Anatomy, National and Kapodistrian University of Athens, Athens, GRC; 3 Anatomy and Surgical Anatomy, Aristotle University of Thessaloniki, Thessaloniki, GRC

**Keywords:** division, trifurcation, bifurcation, superficial brachial artery, variation, axillary artery

## Abstract

The brachial artery (ΒΑ) represents the axillary artery’s extension as it distally progresses to the teres major muscle or beneath the tendon of the latissimus dorsi muscle. Throughout its course, the BA maintains continuous proximity to the median nerve. Occasionally, an artery located in front of the arm muscles may exhibit a slightly more lateral position than the BA, following a convoluted path, referred to as the superficial brachial artery (SBA). SBA variants are not uncommon and can impact neural structures as well. In the course of routine dissection on a formalin-embalmed donated cadaver through the Body Donation Program, the following notable findings were identified: a) a BA bilateral trifurcation, below the tendon of the latissimus dorsi muscle; b) a posterior circumflex humeral artery of low origin (brachial artery); c) the coexistence of an SBA with the main BA; d) a subscapular artery of high origin (second part of the axillary artery); e) an anterior circumflex humeral artery duplication.

These BA variants, particularly those related to the SBA, hold significance in upper limb surgery and everyday clinical practice. In such cases, meticulous surgical dissection is crucial to prevent arterial injury, and in complex situations, preoperative imaging might be advisable. Additionally, it's important to note that concurrent neural variants may also be present, potentially complicating the surgical approach.

## Introduction

The brachial artery (BA) is the axillary artery’s (AA’s) continuation distally to the teres major muscle or below the tendon of the latissimus dorsi muscle (LD). The BA typically gives off the deep brachial artery (DBA, or profunda brachii artery), the nutrient humeral artery, and the superior and inferior ulnar collateral arteries. The BA finally bifurcates, usually in the cubital fossa, into the radial artery (RA) and ulnar artery (UA). The BA along its course has a continuous relationship with the median nerve (MN) [1].

Atypically, the BA may travel anterior to the MN (altered location) or may be divided into a superficial and a deep branch. The BA in its anterior course in relation to the MN roots (axilla) and the MN (arm) is called the superficial brachial artery (SBA). Alternatively, the SBA may first travel posterior to the MN roots and then anterior to the MN, more distally in the arm. The SBA is located anterior to the arm muscles, slightly more lateral than the BA, and follows a tortuous course [1]. The SBA has a prevalence ranging from 1.07% to 19.7% [2,3] and may arise from the AA, or from the BA proximal end, usually between the branches of the medial and lateral cords (MC and LC) of the brachial plexus (BP) [1]. The DBA typically originates from the BA [1]. Przybycień et al. identified the DBA origin from the BA or AA at a high prevalence of 92.87%; however, other variants, such as the DBA origin from the AA or the BA, via a common trunk with other arteries, are not quite uncommon [4]. When the BA is duplicated in the arm or possesses a high bifurcation, the SBA and DBA may distally reunite [5]. SBA may become the RA or the UA with or without anastomoses [5]. The SBA may also become the artery of the MN or the superficial antebrachial artery [6, 7]. In the Natsis et al. report [8], the SBA bifurcated into the RA and UA, as usual, while in other studies, the SBA ended in the arm or continued as RA (high origin of RA) or UA (high origin of UA) with both arteries coursing superficially [9, 10].

In the current cadaveric report, an unusual BA division into superficial and deep trunks and other aberrant vessels fused in the form of a trunk was identified. The BA trunks terminated into RA and UA with or without anastomosis between them. Neurovascular co-variants were also recorded and further discussed.

## Case presentation

**Figure 1:**
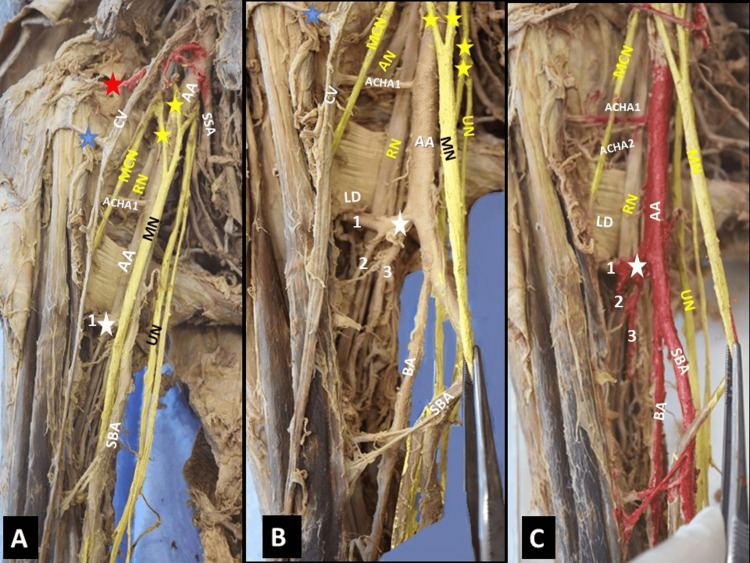
The brachial artery (BA) trifurcation on the right arm, below the tendon of the latissimus dorsi muscle (LD) A. B. The axillary artery (AA) continuation and its trifurcation below the tendon of the LD into a superficial brachial artery (SBA), and the main BA, B. a common trunk (CT) that gave off an aberrant posterior circumflex humeral artery (1), the deep brachial artery (3) accompanied by the radial nerve (RN), muscular branches, and the nutrient humeral artery (2). A. The median nerve (MN) is formed by two lateral roots (yellow asterisks) and a medial root (white asterisk). C. The anterior circumflex humeral artery (ACHA1, ACHA2) duplication. UN-ulnar nerve, AN-axillary nerve, SSA-subscapular artery high origin from the AA second part

During the dissection of an 80-year-old formalin-embalmed donated male cadaver, a bilateral trifurcation of the BA, below the LD tendon, was identified, with an asymmetric termination into the RA and UA, with or without anastomosis between the ending vessels, at the right and left arms. The right-sided AA, at its second part, was divided into a trunk posterior to the atypical-formed MN, and a trunk medial to the MN (Figure 1A). The medial trunk gave off the subscapular artery - SSA (at a higher level than usual, the second part of the AA) and its branches, and the trunk posterior to the MN continued as the main BA. Just after the MN formation, an accessory lateral root - LR (second LR) was identified, thus creating a variant MN of three roots (two LRs and a medial root - MR) (Figure 1B). The AA, on the lower border of the LD tendon, was trifurcated into (1) an SBA of a tortuous and anterior course to the MN, (2) a main BA, posterior to the MN, and (3) a common trunk of the aberrant posterior circumflex humeral artery (PCHA), the DBA, muscular branches, and the nutrient humeral artery (Figure 1C). The SSA of high origin (2nd part of the AA) (Figure 1A) coexisted with a duplication of the anterior circumflex humeral artery (Figure 1C).

**Figure 2:**
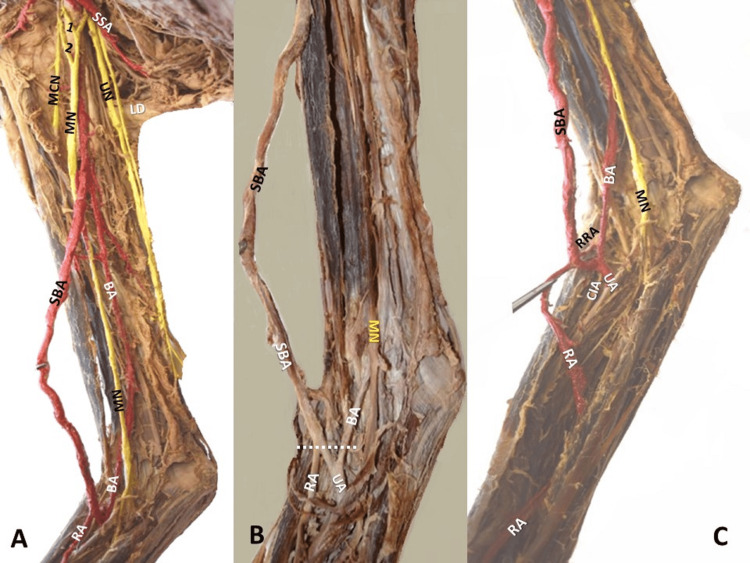
The brachial artery’s (BA's) termination at the right arm A. The BA trifurcation below the tendon of the latissimus dorsi muscle (LD) into a superficial brachial artery (SBA) of a tortuous course (A), the main BA, and the common trunk that gave off the aberrant posterior circumflex humeral artery, deep brachial artery, muscular branches, and nutrient humeral artery. A. The median nerve (MN) is formed by two lateral roots (1,2) and a medial root. B. C. The SBA terminated as the radial artery (RA) and the BA as the ulnar artery (UA) after the anastomosis of SBA and BA (white dotted line). C. RRA-recurrent radial artery, branch of a SBA, CIA-common. interosseous artery, UN-ulnar nerve

At the cubital fossa, the SBA was bifurcated into the RA, an anastomotic branch (length of 15 mm), a recurrent RA, and a muscular branch (Figure 2C). The anastomotic branch was fused to the main BA (Figures 2A, 2B), further giving off the common interosseous artery and the UA (Figure 2C). On the left arm, the AA followed a similar path between the MR and LR of the MN and trifurcated into a tortuous SBA, a main BA, and a common trunk for the aberrant PCHA (a PCHA of low origin from the BA trunk) and the DBA.

**Figure 3:**
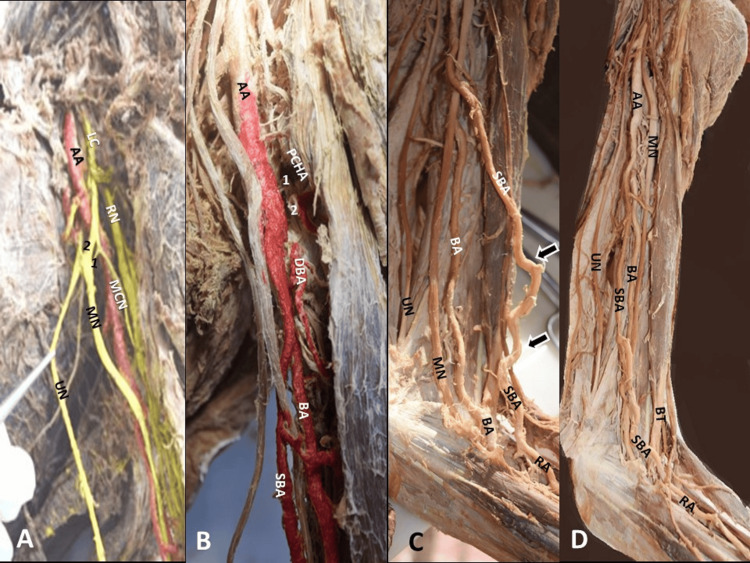
The brachial artery (BA) trifurcation and its covariant, at the left arm A. The atypically formed median nerve (MN) by two lateral roots (1,2) anterior to the axillary artery (AA) course, LC-lateral cord, MCN-musculocutaneous nerve, RN-radial nerve, UN-ulnar nerve, B. The left-sided BA trifurcation into a superficial brachial artery (SBA), a main BA, and a common trunk for the aberrant posterior circumflex humeral artery (PCHA, 1-cut origin)and the deep brachial artery (DBA, 2-cut origin). C. The SBA tortuosity (black asterisks) and course anterior to the median nerve (MN). C, D. At the cubital fossa, the SBA’s termination into the radial artery (RA), and the main BA termination into the ulnar artery (not depicted). D. The SBA’s origin is posterior to the MN, and after its emersion is anterior to the MN, shortly after its origin, and BT-bicipital tendon.

**Figure 4:**
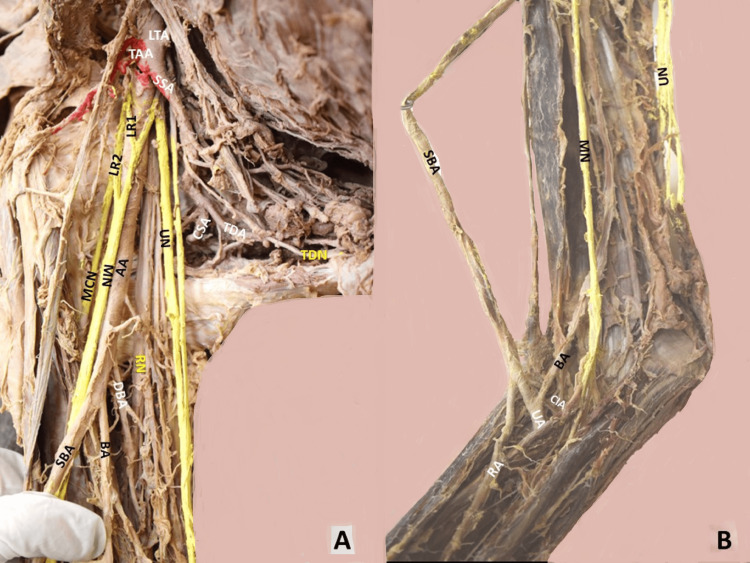
The axillary artery (AA) at the right upper limb. A. The axillary artery’s (AA) continuation below the tendon of the latissimus dorsi muscle and its trifurcation into a superficial brachial artery (SBA), the common trunk (not evident) of (the deep brachial artery- DBA, the posterior circumflex humeral artery and the nutrient humeral artery), and the main brachial artery (BA), at the right arm. The atypically formed median nerve (MN) by two lateral roots (1,2) anterior to the AA course, MCN-musculocutaneous nerve, RN-radial nerve, and UN-ulnar nerve. The AA division after the thoracoacromial and lateral thoracic arteries’ (TAA and LTA) origin into the subscapular artery (SSA) and further branching into the circumflex scapular artery (CSA) and the thoracodorsal artery (TDA), accompanied by the thoracodorsal nerve (TDN). B. The right-sided BA and SBA division after their anastomosis into the radial artery (RA, from the SBA), and the ulnar and common interosseous arteries (UA and CIA) from the BA after its anastomosis with the SBA.

Although the SBA originated posteriorly to the MN, it emerged anterior to the nerve shortly after its origin. Ipsilaterally, a duplication of the LR of the MN was identified, anterior to the AA (Figure 3). At the cubital fossa, the SBA gave off the RA, and the main BA gave off the common interosseous artery and the UA (Figures 2C, 4). The dissection was performed, according to Romane's [11] protocol. Upper limbs were free of any physical deformity or trauma. The cadaver was donated through the “Body Donation Program” after written informed consent (the human ethical approval number is not applicable).

## Discussion

The current case describes an SBA and its coexistence with fused arteries, branches of the AA or BA, as well as the arteries’ migration (PCHA at a lower level and the SSA, at a higher level than usual). Singer described the upper limb arterial variants as remnants of previous embryonic arterial trunks, such as the axial artery that usually regresses after the 8th developmental week [12]. In 2001, Rodríguez-Niedenführ et al. published a new theory, emphasizing that all principal upper limb arterial trunks are formed from a primitive capillary network [10,13]. This theory offers an easy explanation of all reported arterial variants of the upper limb. Arey [14] and Jurjus et al. [5] suggested six explanations for the reported variants: 1. The choice of unusual paths in the primitive vascular plexus, 2. the persistence of vessels that are normally obliterated, 3. the disappearance of vessels that are normally retained, 4. the incomplete development, 5. the fusion and absorption of parts that are normally distinct; and 6. a combination of factors leading to an atypical pattern.

The prevalence of the SBA in different populations is summarized in Table 1.

**Table 1:** The prevalence of the superficial brachial artery (SBA) in different studies, irrespective of its origin

Author(s)	Year	Population/ Country	Sample (specimens’ number)	Incidence (%)
De Garis [15]	1928	American	512	4.6
Adachi [16]	1928	Japanese	1198	3.1
McCormack et al. [2]	1953	American	750	1.07
Skopakoff [3]	1959	-	610	19.7
Keen [9]	1961	African & Swiss	284	3.6
Fuss et al. [17]	1985	German	200	17
Rodriguez-Baeza et al. [18]	1995	Spanish	160	11.9
Rodriguez-Niedefuehr et al. [13]	2001	Spanish & British	112 (embryos)	7.7
Rodriguez-Niedefuehr et al. [10]	2001	British	384	4.9
Yang et al. [19]	2008	Korean	304	12.2
Kachlik et al. [20]	2010	Czech	130	5
Chakravarthi et al. [21]	2014	Indian	140	7.14
Bolden et al. [22]	2020	American	174	5.2

Although SBA is not an uncommon variant (prevalence ranges between 1.07-19.7%), its bilateral presence is quite unusual according to the Rodriguez-Niedenführ et al. study (0.5%, 1 out of 194 cadavers) [10] while Yang et al. recorded the higher prevalence of 6.5% (10/154 cadavers) [19]. Jurgus et al. [5] and Sharma et al. [23] described this variant in their cadaveric reports. Rodriguez-Niedenfuhr et al. mentioned the SBA dominance in males, on the right arm [10]. De Garis and Swartley supported that the SBA is more frequent in African Americans (13.4%) than in Caucasians (4.6%) [15].

In the current case, the DBA originated by a common trunk with the PCHA (occurring at 4% in the Charles et al. study [24]). The DBA was classified by Charles et al. into seven morphological types [24]. The commonest type of DBA was the direct origin from the AA or BA. In the Przybycien et al. meta-analysis [4], the prevalence of the DBA direct origin from the AA or the BA was calculated at 92.87% while the indirect origin by a common trunk was identified at 7.13%, close to the Charles et al. classification [24]. 

In the current series, the BA course was recorded between the MN roots and then divided into SBA (anterior to the MN) and the main BA (posterior to the MN), which was identified in 8% by Tubbs et al. [25]. The most frequent location of the BA is recorded posterior to the MN in 75% [25].

In the current case, two different terminations of the BA were identified. The left-sided SBA gave off the RA, and the main BA gave off the common interosseous artery and the UA. This pattern was identified in 5% [26]. The right-sided SBA gave off the RA and a branch that joined the main BA before it gave off the common interosseous artery and the UA. Lippert and Pabst identified a main BA that bifurcated into UA and a branch that joined the SBA in 3% [26]. The commonest termination of the SBA into the RA and UA was recorded at 70% [25]. Keen [9] identified three SBA morphological types, in relation to their course and division: 1. an SBA that bifurcated into the cubital fossa, 2. an SBA that continued as RA and was called “RA high origin” while Rodriguez-Niedenfuhr et al. [10] proposed the term “superficial brachioradial artery” for its characterization as more appropriate, and 3. an SBA that continued as UA and was called “UA high origin” while Rodriguez-Niedenfuhr et al. [10] proposed the term “superficial brachio-ulnar artery”, as more appropriate.

The bilateral coexistence of the SBA along with the common origin of the DBA with the PCHA (via a common trunk) and the SBA termination into the RA and its anastomosis with the main BA (that ended as UA) is quite unusual. Piagkou et al. described the SBA (of axillary origin) coexistence with an atypical MN (formatted by three roots, two lateral and a medial) and the SBA anastomosis with the main BA (with a posterolateral location to the MN), and both arteries’ recombination before their division into RA and UA [27]. Paraskevas et al. [28] and Natsis et al. [8] identified a coexistence of an SBA with an LR duplication, similar to the current case. In the Natsis et al. report, the right-sided AA, bifurcated into a superficial axillary trunk, anteromedial to the MN and laterally to the ulnar nerve, and a deep axillary trunk that continued as the main BA, laterally to the LR duplication [8]. The superficial trunk continued as the SBA. Both SBA and main BA fused before the complex bifurcation into RA and UA. The LR duplication coexisted with a musculocutaneous nerve duplication. The deep axillary stem provided the posterior and anterior circumflex humeral arteries and descended posteriorly to the MN, then giving off the nutrient humeral artery and a common trunk for the PBA, a deep muscular branch, and a branch to the posterior arm compartment [8]. SBA terminated as the RA and the BA as the UA [8]. The LR duplication has been reported at 4.65% in Pandey and Shukla's study [29].

The BA variants play an important role in upper limb surgery, concerning orthopedic, vascular, as well as plastic surgeons. Although the identification of SBA is not common, it holds significance since it is more prone to injury due to traumatic events or other conditions such as gangrene. In everyday clinical practice, accidental intravenous drug administration may prove lethal while the insertion of a vein catheter may lead to injury and/or thrombosis that could lead to ischemia. Furthermore, anterior approaches to the arm, mainly the expansion of the anterior (deltopectoral) approach for the treatment of humeral fractures may put in jeopardy the SBA, as well as the musculocutaneous nerve in the proximal part of the approach [30]. Meticulous dissection is advised since in such cases that variations are present, they may be noted not only at the vascular but also at the neural elements. The location of the SBA may also be of paramount importance during a free arm or forearm flap and/or coronary bypass surgery. In these cases, further imaging with angiography may be useful.

## Conclusions

BA variants and, more specifically, SBA morphological and/or topographical variants play an important role mainly in upper limb surgery, as well as in everyday clinical practice. Thorough surgical dissection during these approaches is of utmost importance for avoiding arterial injury, while in complex cases, preoperative imaging could be considered. Furthermore, it is also of note that concomitant neural variants may also be present in these cases, which may complicate the surgical approach.
